# Is low-back pain a limiting factor for senior workers with high physical work demands? A cross-sectional study

**DOI:** 10.1186/s12891-020-03643-1

**Published:** 2020-09-21

**Authors:** Patrick Pascal Nygaard, Sebastian Venge Skovlund, Emil Sundstrup, Lars Louis Andersen

**Affiliations:** 1 Musculoskeletal Disorders and Physical Workload, National Research Centre for the Working Environment, Lersø Parkallé 105, 2100 Copenhagen, Denmark; 2 Sport Sciences, Department of Health Science and Technology, Aalborg University, Aalborg, Denmark

**Keywords:** Musculoskeletal disease, Occupational medicine, Ergonomics, Work ability, Physical work, Workplace, Sustainable employment, Low-back, Work limitations

## Abstract

**Background:**

Low-back pain (LBP) is highly prevalent among senior workers and may affect work ability, especially among those with hard physical work. This study determined the joint association of LBP intensity and physical work demands with work limitiations due to pain in senior workers.

**Methods:**

In the SeniorWorkingLife study (2018), 11,738 senior workers (≥50 years) replied to questions about physical work demands, LBP intensity, and work limitations due to pain. Using logistic regression analyses and controlling for potential confounders, associations between the physical work demands and LBP intensity (interaction) with work limitiations due to pain (outcome) was modeled.

**Results:**

Higher LBP intensity, as well as higher physical work demands, significantly increased the odds of experiencing work limitiations due to pain, and these two factors interacted with each other (*p* < 0.0001). In analyses stratified for LBP intensity, higher physical work demands gradually increased the odds of experiencing work limitiations due to pain.

**Conclusions:**

Senior workers with a combination of physically demanding work and LBP are more affected by their pain during everyday work tasks compared to workers with similar LBP-intensity in sedentary occupations. Accommodation of work demands seems especially relevant for this group of workers.

## Background

Low-back pain (LBP) is the greatest cause of disability globally [1–3]. It is estimated that 50–80% of all adults will experience LBP at some point in their life [4, 5], and especially the elderly are at risk of experiencing LBP as the incidence and prevalence increase with age [6, 7]. According to the 2018 Danish Work Environment Cohort Study (DWECS), 32.5% of the Danish workforce between 18 and 65 years of age have been experiencing musculoskeletal pain several times a week during the past 3 months while 5.1% are limited in their job due to pain [7].

In addition to aging, both individual and environmental risk factors for LBP have been identified [8, 9]. Individual factors may include metabolism, biochemistry, physical/anthropometrical factors (e.g. a long back), and depressive symptoms among other things [8]. As for the work environment, both psychosocial factors (e.g. low job satisfaction and collegial support) and physical demands (e.g. manual labour including frequent lifting/handling heavy loads, lengthy periods of standing as well as forward bending of the back) have been associated with an increased risk of LBP [7, 8, 10–13]. Thus, according to the Global Burden of Disease Studies in 2009, the overall burden of LBP arising from mechanical exposures at work is estimated to account for around 21.8 million disability adjusted life years [6]. LBP is associated with lower work ability [14–18], risk of sickness absence [19–23], risk of early retirement/ disability pension [24], and early death [25], all of which affect individuals and the society to a significant extent [26, 27]. A Danish study found that as much as one-fifth of the study sample with LBP and neck-shoulder (NS) pain experienced long-term sickness absence (≥3 weeks) within 2 years, with pain intensity and heavy physical work being the main prognostic factors [28]. In addition, a 2005 systematic review on prognostic factors for duration of sick leave, identified LBP and heavier work as primary predictors for a longer duration of sick leave [13]. Studies have suggested that higher pain intensities in combination with physically demanding work may be especially detrimental for the ability to work [28]. Whereas some workers with LBP are highly limited in their job duties, other workers with LBP are not as affected and limited in their work [13, 28]. Thus, a worker with severe LBP undertaking an office-job may be less limited in his/her work duties than workers with a less painful LBP having a physically demanding manual labour. Identifying factors explaining this discrepancy could help tailor effective solutions at the workplace to reduce and prevent LBP and its associated work limitations.

Furthermore, it is estimated that the proportion of older workers (50–64 years of age) will increase considerably in the near future [29]. Granted the increased prevalence and incidence of LBP with age and its associated negative consequences, it is critical to identify sustainable ways of employing through aging.

The study aimed to estimate the joint association of LBP intensity and physical work demands with work limitations due to pain among senior workers. We hypothesized that high physical work demands would aggravate the association between LBP intensity and work limitations.

## Methods

### Study design and setting

This study employs data on work limitiations due to pain and physical demands from the 2018 round of the SeniorWorkingLife study (SWL). SWL is a Danish questionnaire-survey covering 14 domains in relation to push and stay mechanics for labour market participation among the elderly [30]. The baseline questionnaire was sent out in July 2018 and baseline data collection was terminated in October 2018. The SWL aspire to do long-term follow-up every 2–3 years using Danish national registers and surveys. SWL is registered as a cohort study in ClinicalTrials.gov (Identifier: NCT03634410) [30].

### Participants

At baseline, 30,000 Danes aged 50 years or older were invited to participate in the SWL questionnaire survey of which 18,000 were employed, 7000 unemployed, 3000 on voluntary early retirement, and 2000 on disability pension [30]. The invited participants were drawn as a probability sample by Statistics Denmark and the questionnaire was sent through E-boks [30], which is a secure Danish mailing system linked to the social security numbers [31]. For the present analysis, only currently employed workers were included. Among those, 56% replied to the entire questionnaire survey, however, we also included workers who only replied partly to the questionnaire, yielding a total study sample of 11,738 senior workers (~ 65% of 18,000). The baseline characteristics of the study population can be seen in Table 1.

**Table 1:** Baseline characteristics of the sample

	***n***	Mean (95% CI)	SD	% (95% CI)
**Age (years)**	12,879	56.6	5.4	
**Sex, men/women**	7054/5825			53.4/46.6
**BMI (kg/m**^**2**^**)**		26.4	5.1	
**Smoking**				
**No, never**	5714			48.3 (47.3–49.3)
**Ex-smoker**	4110			34.3 (33.3–35.2)
**Yes, but not every day**	373			3.3 (2.9–3.6)
**Yes, every day**	1729			14.2 (13.5–14.9)
**Physical activity during leisure**				
**Mostly sedentary**	1779			14.8 (14.0–15.5)
**Light exercise at least 4 h**	7202			60.9 (59.9–61.9)
**Sports or heavy physical activity at least 4 h per week**	2697			22.3 (21.5–23.1)
**Training and competing regularly and several times a week**	233			2.0 (1.7–2.3)
**Psychosocial work factors (0–100)**				
**Support/recognition from colleagues**	12,111	77.0 (76.6–77.4)	22.5	
**Influence at work**	12,128	77.5 (77.1–77.9)	23.8	
**Physical activity at work**				
**Mostly sedentary work that is not physically demanding**	5909			47.4 (46.3–48.4)
**Mostly standing and walking work that is otherwise not physically demanding**	2698			23.6 (22.7–24.4)
**Standing or walking work with some lifting- and carrying tasks**	2779			22.9 (22.0–23.8)
**Heavy or fast work that is physically demanding**	787			6.2 (5.7–6.7)

*BMI* Body mass index (kg/m^2^), *n* number, *SD* Standard deviation; *%* percentage

### Ethical considerations

According to Danish law, questionnaire and register-based studies do not require approval by ethical and scientific committees, nor informed consent [32, 33]. All data were de-identified by Statistics Denmark and remained on the server of Statistics Denmark from where it was analyzed through remote access by the researchers [30].

## Explanatory variables

### Physical work demands

Participants replied to the following question to determine the participants’ physical work demands: ‘How would you describe the physical activity level in your current job?’. The four response options were: 1) ‘Mostly sedentary work that is not physically demanding’ 2) ‘Mostly standing and walking work that otherwise is not physically demanding’, 3) ‘Standing or walking work with some lifting and carrying tasks’ and 4) ‘Heavy or fast work that is physically demanding’.

### Musculoskeletal pain

Participants were asked to report their average pain intensity for the low-back during the past 3 months on a 0–10 scale, with 0 being no pain and 10 indicating worst imaginable pain. For further analyses, the participants were divided into the following groups based on their LBP-intensity: ‘No or little pain’ (pain intensity 0–2), ‘Moderate pain’ (pain intensity 3–4), ‘High pain’ (pain intensity 5–6), ‘Very high pain’ (pain intensity ≥7).

### Outcome variables

To assess work limitation due to LBP, participants replied to the following question: ‘To which degree did the pain limit you in your work during the last 3 months?’, with a response scale of 1) ‘to a very high degree’, 2) ‘to a high degree’, 3) ‘to some degree’, 4) ‘to a lesser degree’ and 5) ‘not at all’. Work limitation due to pain was further dichotomized in to ‘No or to a small degree’ (the first two response options) and ‘Yes, to some or a very high degree’ (the last three response options together), respectively.

### Control variables

In the present study, we controlled for multiple potential confounders. The control variables were selected since they have previously been associated with pain, work limitations, and/or physical activity at work [34–41]. The analyses were controlled for age (continuous scale, years), sex (categorical; ‘male’ or ‘female’), smoking status (categorical; ‘No, never’, ‘Ex-smoker’, ‘Yes, but not every day’ and ‘Yes, every day’), body mass index (BMI) (continuous scale; kg/m^2^), musculoskeletal pain in the neck/shoulder, arms and legs (continuous scale 0–10), psychosocial work environment (described below), educational level (described below), and physical activity during leisure (described below).

The psychosocial work environment was assessed on a 0–100 scale by questions regarding collegial recognition and influence at work originating from the Copenhagen Psychosocial Questionnaire [42]. Physical activity during leisure was assessed by the following question: ‘How would you describe your physical activity level during leisure for the last 12 months?’ with four different response options: ‘Mostly sedentary, ‘Light exercise at least 4 h per week’, ‘Sports or heavy physical activity at least 4 h per week’ and ‘Training and competing regularly and several times a week’. Educational level was assessed by Danish register data on highest completed educational level: 1) Primary school or unknown, 2) High school, 3) Short-term higher education, 4) Medium-term higher education, and 5) Long-term higher education.

### Statistical analysis

Using logistic regression (Proc Glimmix, SAS version 9.4, SAS Institute, Cary, NC), we modelled the association between LBP intensity and physical work demands (predictors), as well as the interaction between these, with the odds of experiencing work limitiations due to pain (outcome). Model 1 (minimally adjusted) was adjusted for age, sex, and pain in the other body regions. Model 2 (fully adjusted) was adjusted for model 1 as well as smoking status, body mass index (BMI), psychosocial work environment, educational level, and physical activity level during leisure. Model-assisted statistical weights including sex, age, occupational industry, highest completed education, family income, family type, and origin were used to make the estimates representative. The results are reported as ORs and 95% confidence intervals (CI) stratified for LBP intensity as there was a significant interaction between LBP intensity and physical work demands. An alpha level below 0.05 was chosen as statistically significant differences.

## Results

### Baseline characteristics

Table 1 shows the baseline characteristics of the study sample. The mean age of the sample was 56.6 years with 47% holding jobs characterized as ‘Mostly sedentary work that is not physically demanding’, 24% as ‘Mostly standing and walking work that otherwise is not physically demanding’, 23% as ‘Standing or walking work with some lifting- and carrying tasks’ and 6% as ‘Heavy or fast work that is physically demanding’. During the last 3 months, approximately 35% of the sample experienced pain in low-back whereas 13.3% of the sample reported limitations due to pain to some degree, 3% to a large degree, and 1.2% to a very large degree.

### Physical work demands and work limitiations due to low-back pain

**Fig. 1:**
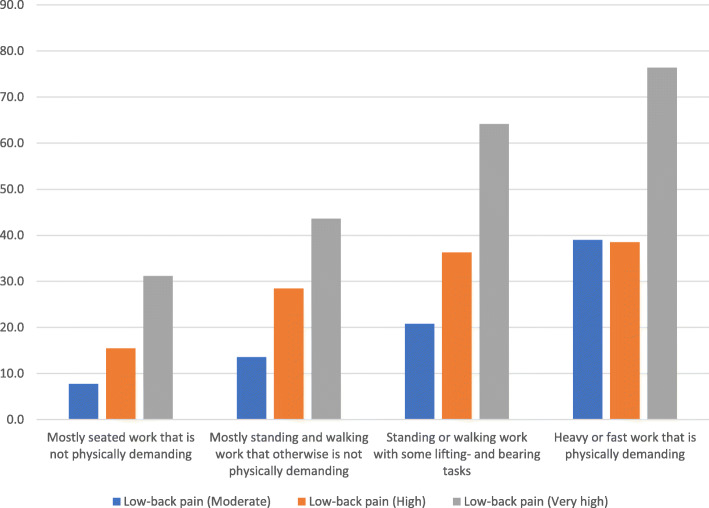
Weighted prevalences of work-limiting pain by physical work demands and low-back pain intensity

Figure 1 shows the weighted prevalences of work limitiations due to pain by physical work demands and LBP intensity. Table 2 shows the associations between physical work demands and work limitiations due to pain stratified by LBP intensity.

**Table 2:** Odds ratios (OR) and 95% confidence intervals (95% CI) for physical work demands and work limitations due to pain stratified by low-back pain intensity among senior workers. The reference group is "Mostly seated work that is not physically demanding"

	Physical work demands	Model 1	Model 2
Moderate pain (3–4)	Mostly standing and walking work that otherwise is not physically demanding	1.79 (1.70–1.88)	1.82 (1.73–1.92)
	Standing or walking work with some lifting and carrying tasks	2.72 (2.60–2.84)	2.60 (2.48–2.73)
	Heavy or fast work that is physically demanding	5.04 (4.72–5.38)	4.61 (4.31–4.94)
High pain (5–6)	Mostly standing and walking work that otherwise is not physically demanding	2.32 (2.22–2.42)	2.24 (2.14–2.34)
	Standing or walking work with some lifting and carrying tasks	2.59 (2.50–2.69)	2.41 (2.32–2.51)
	Heavy or fast work that is physically demanding	2.39 (2.26–2.52)	2.17 (2.05–2.30)
Very high pain (7–10)	Mostly standing and walking work that otherwise is not physically demanding	1.49 (1.43–1.56)	1.43 (1.36–1.49)
	Standing or walking work with some lifting and carrying tasks	3.00 (2.88–3.12)	2.70 (2.59–2.81)
	Heavy or fast work that is physically demanding	4.33 (4.10–4.58)	3.56 (3.36–3.76)

Model 1 (minimally adjusted): Adjusted for age, sex, and pain in the other body regions. Model 2 (fully adjusted): Adjusted for model 1 as well as smoking status, body mass index (BMI), psychosocial work environment, educational level, and physical activity level during leisure

We found a significant dose-response relation between LBP intensity and work limitiations due to pain (*p* < .0001) and between physical work demands and work limitiations due to pain among senior workers (*p* < .0001). The interaction between LBP intensity and physical work demands in relation to work limitiations due to pain was significant (*p* < .0001). The results indicate that the prevalence of work limitiations due to pain increases with higher back pain intensity and heavier/higher work demands. Thus, the weighted prevalence among workers reporting a combination of very high LBP intensity and ‘heavy or fast work that is physically demanding’ (being the most exposed group of workers) is approximately 76%. In comparison, the prevalence in the group reporting the same physical work demands but experiencing high pain instead of very high is around 38%. For the group reporting very high LBP but a less physically demanding job (mostly sedentary work that is not physically demanding), the prevalence is down to approximately 31%.

Table 2, illustrates a general increase in odds for work limitations due to pain with higher work demands and pain intensity. As an example in model 1, we observed significantly increased odds of work limitiations due to pain when comparing the reference group to workers with moderate pain (3–4) and a ‘mostly standing and walking work that otherwise is not physically demanding’ (OR: 1.79, 95% CI: 1.70–1.88) (Table 2). Even stronger associations are seen in workers with the same LBP intensity but with ‘Standing or walking work with some lifting- and carrying tasks’ (OR: 2.72, 95% CI: 2.60–.84) and in workers with ‘heavy or fast work that is physically demanding’ (OR: 5.04, 95% CI: 4.72–5.38).

## Discussion

Our study shows that the combination of high physical work demands and high LBP intensity markedly increases the odds of work limitations due to pain. Thus, senior workers with a combination of physically demanding work and LBP seem to be more affected by their pain during work compared to workers with similar LBP-intensity in sedentary employment. Accommodation of work demands an implementation of workplace exercising seems especially relevant for this group of workers.

### Low-back pain prevalence, intensity and work limitations due to low-back pain

This study found a significant dose-response relation between LBP intensity and work limitiations due to pain (*p* < .0001). Previous studies have reported similar findings. These studies have employed outcomes such as reduced work ability and work performance, as well as productivity loss, as measures for work limitations. Among these, a 2012 cross-sectional study reported that most workers with chronic nonspecific musculoskeletal pain experience poor to moderate work ability and work performance [24]. Other studies on adults (20–60, 31–59 and 24–69 years of age in the three studies) suggest associations between having LBP and work ability and performance as well as productivity loss [43–45]. One of these studies found that especially workers aged 47 or older had a reduced work ability [44]. Furthermore, studies have shown that work ability is negatively affected by pain intensity [46] and by pain in multiple sites of the body [15], while other studies showed that pain located in only one body region is well enough to affect work ability in a negative way [17, 47].

According to the findings of a 2019 review, fear of musculoskeletal pain reoccurrence and the following avoidance of certain movements (fear-avoidance beliefs) is one of the main reasons why LBP negatively affects work ability (other than experiencing pain) [16]. Other than work ability pain related fear appears to be associated with presenteeism [48]. This underlines that musculoskeletal pain and its relation to work ability is complex and multifactorial. Studies suggest that fear of pain or re-injury can be significantly reduced, using cognitive-behavioral therapy [49] or highly individualized and tailored exposure therapy or education [50].

Aside from pain affecting work ability, several studies indicate an association with sickness absence as well. Holtermann et al. (2010) showed that pain intensity was one of the main risk factors for increased sickness absence among workers with LBP and/or neck-shoulder pain [28], and several other studies on adults (mean age 45 (SD = 10), 46 (SD = 9.5), 49.63 (SD = 9.71)) have reported similar findings [14, 17, 18, 47]. Thus, our study results are compatible with several other studies investigating LBP and pain in other body regions, indicating that the evident negative impact of higher pain intensity on work limitation is not limited to the low-back but is also transferable to MSD/pain in other body regions.

### Heavy physical work and work limitiations due to low-back pain

In addition to showing an association between pain intensity and work limitations, this study also showed that high physical work demands aggravate the association of LBP-intensity with work limitations due to pain. This is in accordance with previous studies reporting lower work ability among workers with MSD and high physical work demands [51–54], while high physical work demands also seem to increase sickness absence among workers with MSD [28, 55–61].

Thus, it seems that seniors with physically demanding work and LBP are more affected by their pain during everyday work tasks compared to workers with similar LBP-intensity undertaking sedentary employment. Solutions and effective measures to reduce LBP and its consequences, therefore, seem to be especially important for workers with physically demanding work. Better fitting the work task to the capability of the seniorworker (e.g. by decreasing physical demands and introducing technical aids) and increasing physical capacity (e.g. by workplace strength training) seem as potent tools to reduce the consequence of LBP among workers with physical demanding work.

A systematic review from 2014, including six high-quality studies and four low-quality studies concluded that strength exercises with intensity of 70–85% of RM performed in the workplace, three times a week for 20 min are able to reduce musculoskeletal pain in shoulders, wrists, cervical, thoracic and lumbar spine [62]. Furthermore, the study showed that non-specific exercises (stationary biking, stretching, pilates/ relaxation exercises, and plyometric paddling devices and kaiake) also promoted a decrease of pain [62]. A recent Danish systematic review had similar findings and concluded that implementing strength training at the workplace can reduce MSD among workers with physically demanding employment [63].

Another review found a small positive effect of individual-focused workplace interventions (exercise programs among other interventions) on work ability. However, the authors noted that the quality of the evidence base is only moderate and that further high-quality studies are needed [64]. In addition to preventing and treating pain by exercising, several studies suggest lowering work demands and heavy lifting (to achieve a balance between work requirements and work capacity) in order to reduce the risk of LBP [8, 10–13, 65–67]. Workplaces should take into account that workers with pain and high physical work demands may be especially in need of good assistive devices to do reduce the physical workload. While this applies to all workers with high physical work demands, the need seems to be especially high for workers in pain to ensure that their pain does not work limit work performance.

### Strengths and limitations

This study has both strengths and limitations. A strength of the study is that Statistics Denmark drew a probability sample among all eligible Danish residents ≥50 years, which ensured that the data was representative of senior workers in Denmark. The large sample size and the high response rate increases the statistical power and reduces the risk of statistical type II errors. However, selection bias could still have influenced the present results, and since the data is based on questionnaire replies, self-report bias could also have affected the estimates. Further, self-reports can lead to common method variance where e.g. the participant’s general health, mood, and socioeconomic status can affect the answers on physical work demands and pain intensity [68]. The cross-sectional design of the study is a limitation since it does not allow for causal inference. The adjustment for register-based educational level is a strength of the study that eliminated any self-report bias. Even though the remaining of the control variables were based on questionnaire replies, the adjustment for multiple health-, work environment- and lifestyle factors increased the likelihood of estimating the true association of physical work demands and LBP with work limitations due to pain. However, this adjustment for potential confounders (see Table 2) only changed the odds-estimates to a very small degree which may indicate that factors such as education, smoking, BMI, physical exercise, and psychosocial working environment (influence and recognition) play only a minor role in the association of pain intensity and physical work demands with work limitations due to pain. A limitation to the study is that the analyses were not adjusted for chronic diseases or comorbidities. Poor health has previously been associated with work limitations, and not accounting for this could have led to residual confounding. On the other hand, the analyses were adjusted for both lifestyle factors (i.e. smoking and BMI) and educational attainment, that also associates with poor health. Thus, adjusting for multimorbidity could potentially have led to an overadjustment. Further, our outcome measure relates to work limitation due to back pain and not work limitation in general. Still, our results could have been influenced by residual.

The generalizability of the study applies to currently employed senior workers (mean age 56.6). A strength of the study is that Statistics Denmark drew a probability sample among all eligible Danish residents ≥50 years, which ensured that the data was representative of senior workers in Denmark. It should, however, be mentioned, that a study sample closer to state pension age (i.e. 65 years at the point of data collection in 2018) might have influenced the results since pain and work limitations increases with age. However, the statistical models were adjusted for age, inherently accounting for any age-related differences within the study sample.

Finally the relatively small number of workers doing ‘heavy or fast work that is physically demanding’ (*n* = 787) might represent minor limitations in this study.

## Conclusion

The combination of high physical work demands and high LBP intensity markedly increases the odds of work limitations due to pain. Senior workers with a combination of physically demanding work and LBP seem to be more affected by their pain during work compared to workers with similar LBP-intensity in sedentary employment.

## Data Availability

The authors encourage collaboration and use of the data by other researchers. Data are stored on the server of Statistics Denmark, and researchers interested in using the data for scientific purposes should contact the project leader Prof. Lars L. Andersen, lla@nfa.dk, who is responsible for the study design, questionnaire development, definition of population, and data collection.

## Abbreviations

95% CI: 95% confidence intervals
BMI: Body mass index
OR: Odds ratio
MSD: Musculoskeletal disorders
LBP: Low-back pain
